# Technology Acceptance of Home-Based Cardiac Telerehabilitation Programs in Patients With Coronary Heart Disease: Systematic Scoping Review

**DOI:** 10.2196/34657

**Published:** 2022-01-07

**Authors:** Hadassah Joann Ramachandran, Ying Jiang, Jun Yi Claire Teo, Tee Joo Yeo, Wenru Wang

**Affiliations:** 1 Alice Lee Centre for Nursing Studies, Yong Loo Lin School of Medicine National University of Singapore Singapore Singapore; 2 Cardiac Rehabilitation, Department of Cardiology National University Heart Centre Singapore Singapore

**Keywords:** technology acceptance, coronary heart disease, home-based, telerehabilitation, web-based, mobile application, acceptance, heart, rehabilitation, app, review, evaluation, cardiac, cardiology, perspective, usability, acceptability

## Abstract

**Background:**

An understanding of the technology acceptance of home-based cardiac telerehabilitation programs is paramount if they are to be designed and delivered to target the needs and preferences of patients with coronary heart disease; however, the current state of technology acceptance of home-based cardiac telerehabilitation has not been systematically evaluated in the literature.

**Objective:**

We aimed to provide a comprehensive summary of home-based cardiac telerehabilitation technology acceptance in terms of (1) the timing and approaches used and (2) patients’ perspectives on its usability, utility, acceptability, acceptance, and external variables.

**Methods:**

We searched PubMed, CENTRAL, Embase, CINAHL, PsycINFO, and Scopus (inception to July 2021) for English-language papers that reported empirical evidence on the technology acceptance of early-phase home-based cardiac telerehabilitation in patients with coronary heart disease. Content analysis was undertaken.

**Results:**

The search identified 1798 studies, of which 18 studies, with 14 unique home-based cardiac telerehabilitation programs, met eligibility criteria. Technology acceptance (of the home-based cardiac telerehabilitation programs) was mostly evaluated at intra- and posttrial stages using questionnaires (n=10) and usage data (n=11). The least used approach was evaluation through qualitative interviews (n=3). Usability, utility, acceptability, and acceptance were generally favored. External variables that influenced home-based cardiac telerehabilitation usage included component quality, system quality, facilitating conditions, and intrinsic factors.

**Conclusions:**

Home-based cardiac telerehabilitation usability, utility, acceptability, and acceptance were high; yet, a number of external variables influenced acceptance. Findings and recommendations from this review can provide guidance for developing and evaluating patient-centered home-based cardiac telerehabilitation programs to stakeholders and clinicians.

## Introduction

Within the spectrum of cardiovascular diseases, coronary heart disease is the most common cause of mortality and morbidity globally and presents a major health care burden [1]. Cardiac rehabilitation is a widely accepted treatment modality for secondary prevention of coronary heart disease [2], but long-standing challenges regarding accessibility to cardiac rehabilitation facilities, conflicting work and care responsibilities, low socioeconomic status, and costs of rehabilitation programs have led to disappointingly low reported uptake rates among eligible patients worldwide (10% to 30% [3]). A recent challenge is the COVID-19 pandemic [4]. In the acute phase of the pandemic, nonurgent outpatient services, such as center-based cardiac rehabilitation, were partially or completely closed as limited resources and personnel were redirected to critical areas. Even in the long-term phase of the pandemic, efforts to limit the spread of COVID-19 infection through measures such as safe distancing further limited the capacity for delivery of center-based cardiac rehabilitation group exercise and therapy sessions [5]. Thus, alternative secondary prevention strategies for coronary heart disease are a priority across health care systems during the COVID-19 pandemic and beyond [4].

Home-based cardiac telerehabilitation—defined as the use of information and communication technologies (eg, mobile- and web-based platforms, wearable sensor devices) to deliver remote exercise supervision, education, counseling on cardiovascular risk factor modification, and psychosocial support exclusively at home—is one such emerging alternative [6]. A recent systematic review and meta-analysis [7] of randomized controlled trials comparing home-based cardiac telerehabilitation to center-based cardiac rehabilitation in patients with coronary heart disease found equivalent effects on functional capacity, cardiac-related hospitalization, physiological risk factor control, quality of life, depression, and behaviors such as physical activity, smoking cessation, and medication adherence. However, adapting digital solutions for health problems is not without its challenges; attempts to scale up effective digital health research interventions into real-world health care systems have been met with difficulty, especially for complex interventions that require user interaction [8,9]. The successful incorporation of such digital health technologies into clinical practice is contingent upon end-users’ (ie, patients) acceptance and sustained engagement with the intervention, and thus, these are important aspect for researchers, health care systems, and policymakers to consider [9,10].

The technology acceptance model provides a framework for modeling end-user acceptance [11] and theorizes that both perceived usefulness (ie, utility) and perceived ease of use (ie, usability) of a target system directly influence intention to use (ie, acceptability), which then influences actual system use (ie, acceptance of the system) [11]. External variables such as technology self-efficacy and training, objective system design features, and the process of system implementation are thought to indirectly influence system acceptability and acceptance by influencing system utility and usability [11]. An understanding of the usability and utility of home-based cardiac telerehabilitation programs is paramount if they are to be designed and delivered to target the needs of patients with coronary heart disease in a way that ensures programs are accepted. However, the current state of technology acceptance of home-based cardiac telerehabilitation has not been systematically evaluated in the literature. We aimed to provide a comprehensive summary of the technology acceptance of home-based cardiac telerehabilitation among patients with coronary heart disease.

## Methods

### Study Design

We performed a systematic scoping review to comprehensively collate, summarize, and map [12,13] existing evidence on home-based cardiac telerehabilitation research in terms of usability, utility, acceptability, and acceptance testing. We used the Arksey and O’Malley methodological framework [12]: identifying the research questions, identifying relevant studies, study selection, charting the data, collating, summarizing, and reporting the results. To ensure quality and transparency, this review was conducted and reported in accordance with Preferred Reporting Items for Systematic Reviews and Meta-analyses Scoping Review guidelines [14]. A review protocol (not registered) was prepared prior to the start of this review.

### Identifying the Research Question

The following research questions were identified to answer the objective of this review: (1) What are the timing and approaches used to evaluate the technology acceptance attributes in home-based cardiac telerehabilitation? (2) What are patients’ perspectives on the technology acceptance constructs (ie, usability, utility, acceptability, acceptance, and external variables) of home-based cardiac telerehabilitation?

### Identifying Relevant Studies

We followed recommendations by Arksey and O‘Malley [12] and undertook an iterative approach, through ongoing consultations with a university resource librarian throughout the search process, to identify relevant literature. We piloted an initial search strategy in PubMed and EMBASE to identify a sample of relevant papers. This was followed by an analysis of the keywords used in the titles and abstracts and in the indexing of these relevant papers. Preliminary results revealed that terms related to the concept *acceptance* were not commonly indexed in relevant papers. Thereafter, we used terms related to coronary heart disease, rehabilitation, and telehealth. We searched PubMed, Cochrane Central Register of Controlled Trials, Embase, Cumulative Index to Nursing and Allied Health Literature, PsycINFO, and Scopus databases (inception to July 2021). No limits on study design were placed. Additionally, we manually searched the reference lists of relevant systematic reviews and papers included in this review (Table S1 in Multimedia Appendix 1).

### Study Selection

#### Overview

Literature evaluating the technology acceptance constructs of home-based cardiac telerehabilitation that used empirical methods (both quantitative and qualitative) and were published in English were considered. Case reports, conference abstracts, editorials, protocols, and reviews were excluded. The PCC (Population, Concept, Context) framework [14] was used to develop and set the inclusion and exclusion criteria. Search results were imported to Endnote (version X9, Clarivate Analytics) for management. Two independent authors were involved in the study selection process. Records deemed relevant by both authors were included. Consultation with a third author was used to resolve any disagreements regarding inclusion.

#### Population

Papers with a study population of patients with a documented medical diagnosis of coronary heart disease, acute coronary syndrome, myocardial infarction, angina pectoris, or who had undergone revascularization (ie, coronary artery bypass grafting or percutaneous coronary intervention) were included. We excluded papers with a study population of patients with heart failure (regardless of left ventricular ejection fraction), as their therapeutic needs and subsequent evaluations of home-based cardiac telerehabilitation in terms of usability, utility, acceptability, acceptance, and external variables would differ considerably from those of patients with coronary heart disease.

#### Concept

For the purpose of this study, the constructs of the technology acceptance model were conceptualized as follows: (1) usability—degree to which the system is easy to use and free of effort; (2) utility—degree to which the system improves user’s performance and functions as intended; (3) acceptability—behavioral intention or willingness to use the system; and (4) acceptance—actual usage of the system [11,15]. Home-based cardiac telerehabilitation was defined as any mobile health app or website used either as a stand-alone platform or supplemented with other modes of delivery, such as telephone or video calls, short message service, email, or telemonitoring, to exclusively deliver early cardiac rehabilitation or secondary prevention [6]. The decision to focus on mobile- or web-based home-based cardiac telerehabilitation was made with the purpose of scoping the technologies that allowed for greater interaction, flexibility, and independence in rehabilitation programs. Papers were included if they addressed the testing and evaluation of technology acceptance constructs from patient perspectives. Late-phase home-based cardiac telerehabilitation programs, in which the focus is placed on long-term maintenance of lifestyle change, were excluded since we were only interested in the early and active rehabilitation phase (ie, focus on health behavior change, risk factor modification and psychosocial well-being.).

#### Context

The context for telerehabilitation programs was limited to those in a home setting only; hence, we excluded home-based cardiac telerehabilitation delivered alongside center-based cardiac rehabilitation (ie, hybrid cardiac rehabilitation services).

### Charting the Data

Authors, publication year, country of origin, study design, subcategory of coronary heart disease population, sample size, characteristics of home-based cardiac telerehabilitation program, approach, and timing of technology acceptance evaluation data were extracted by the first author and confirmed by the second author, who made adjustments and included additional information where necessary. Features of the home-based cardiac telerehabilitation programs were categorized according to recommendations by Whitelaw et al [16] to facilitate uptake of digital health interventions. We categorized the core components present in the home-based cardiac telerehabilitation programs using American Heart Association classifications [6].

### Collating, Summarizing, and Reporting the Findings

We used a 3-phase process [17] to systematically conduct our content analysis of the technology acceptance of home-based cardiac telerehabilitation among patients with coronary heart disease: preparing, organizing, and reporting of data. Because the aim of the review was to structure a descriptive analysis of home-based cardiac telerehabilitation acceptance based on the constructs of the technology acceptance model, we used a deductive content analysis approach [18,19].

A structured categorization matrix was prepared based on the constructs of the technology acceptance model: usability, utility, acceptability, acceptance, and external variables. Two authors concurrently and independently reviewed all the studies for content and coded data that corresponded to categories in the matrix. Content that did not fit into the other categories was gathered and coded under the category *external variables* and was analyzed based on the principle of inductive content analysis—open coding was undertaken, and data were grouped into similar categories and labeled with subcategories using content-characteristic words [17]. After data were organized, each author reviewed all of the studies under each category to check the reliability of the content analysis process and identify discrepancies in the collating and categorization of study data. Discussions were held until both authors were in agreement with the content under each category. Consultation with a third author was used to resolve any disagreements. The timing of home-based cardiac telerehabilitation evaluations was categorized based on when evaluation was undertaken relative to the trial implementation stage—pretrial, intratrial, or posttrial.

Quality appraisal was not performed as the objective of this scoping review was to provide an overview of the existing evidence on the evaluations of usability, utility, acceptability, and acceptance in home-based cardiac telerehabilitation, regardless of the quality of the evidence [12].

## Results

### General

**Figure 1:**
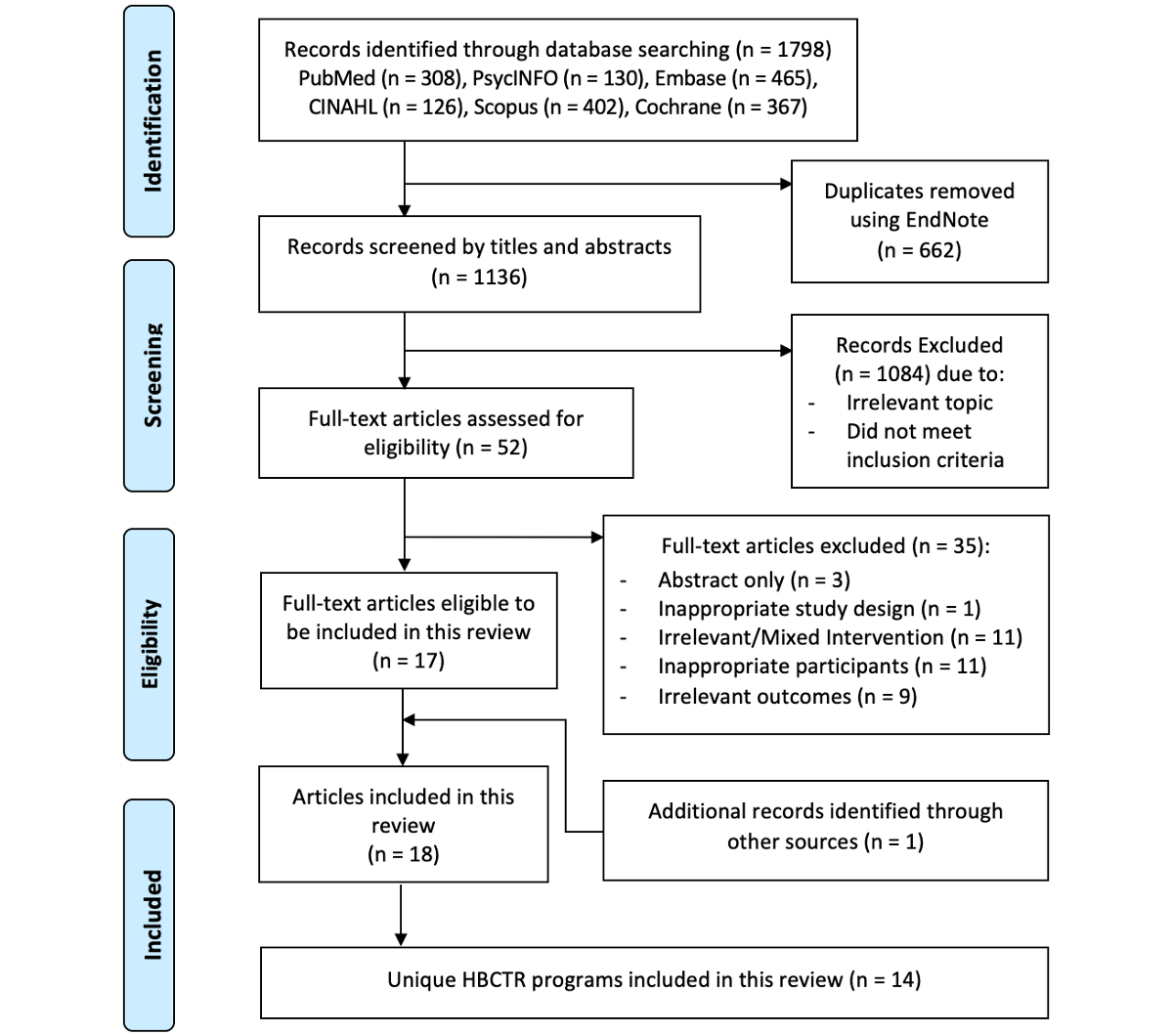
PRISMA (Preferred Reporting Items for Systematic Reviews and Meta-analyses) flow diagram. HBCTR: home-based cardiac telerehabilitation.

The search generated 1136 unique papers. After title and abstract screening, 1084 papers were excluded. The remaining 52 full-text papers were retrieved and screened, and 35 papers were excluded (Table S2 in Multimedia Appendix 1). Manual searches of the reference lists of relevant papers identified 1 paper for inclusion; therefore, 18 papers [20-37], with 14 independent home-based cardiac telerehabilitation programs, were included in this review (Figure 1).

### Characteristics of Studies

Studies included in this review (Table S3 in Multimedia Appendix 2) were published between 2007 and 2021; the majority (n=14) were published after 2013. Studies were conducted in the following countries: China [23,24,28,31,33,34]; Australia [29,30,32,36]; Canada [35-37]; United States of America [22,25]; United Kingdom [20,21]; and New Zealand [27]. Studies included patients who had the following: stable angina; myocardial infarction; stable coronary heart disease; or underwent coronary revascularization (ie, coronary artery bypass grafting or percutaneous coronary intervention). In studies that reported age and gender of participants, the mean age of patients ranged from 53 to 66 years and the proportion of female patients ranged from 9.4% to 33%. Devi et al [21] and Varnfield et al [29] were earlier papers reporting on the same home-based cardiac telerehabilitation programs as those in Devi et al [20] and Varnfield et al [30], respectively. Zutz et al [35] and Lear et al [36] were both earlier papers reporting on the same home-based cardiac telerehabilitation program as that in Banner et al [37].

### Characteristics of Home-Based Cardiac Telerehabilitation Programs

Home-based cardiac telerehabilitation programs were delivered mainly via smartphone apps (n=11) and websites (n=3) and were supplemented by other modes of delivery: text messaging (n=6), telephone calls (n=5), emails (n=2), videoconferencing (n=1), and telemonitoring (n=10). Telemonitoring devices that supported remote supervision of exercise training by the cardiac rehabilitation team and patients’ self-monitoring of physical activity included heart rate monitors, accelerometers, and pedometers.

Features of the home-based cardiac telerehabilitation programs included engagement of stakeholders, clinicians, and patients throughout the design or development of the home-based cardiac telerehabilitation program (n=3); testing of the home-based cardiac telerehabilitation program by cardiology experts and patients (n=8); provision of face-to-face training on use of home-based cardiac telerehabilitation for patients (n=10); ongoing technical support throughout home-based cardiac telerehabilitation program (n=4); and consideration of data privacy and security in the use of technologies in home-based cardiac telerehabilitation (n=7).

The American Heart Association core components [6] that were present in the home-based cardiac telerehabilitation programs were patient assessment (n=14), exercise training (n=13), dietary management (n=10), risk factor management (n=11), medication adherence (n=8), and psychosocial support (n=6). Only 5 studies [23,24,26,30,31] had a comprehensive home-based cardiac telerehabilitation program that included all the core components (Table S4 in Multimedia Appendix 2).

### Timing and Approaches to Evaluation

**Figure 2:**
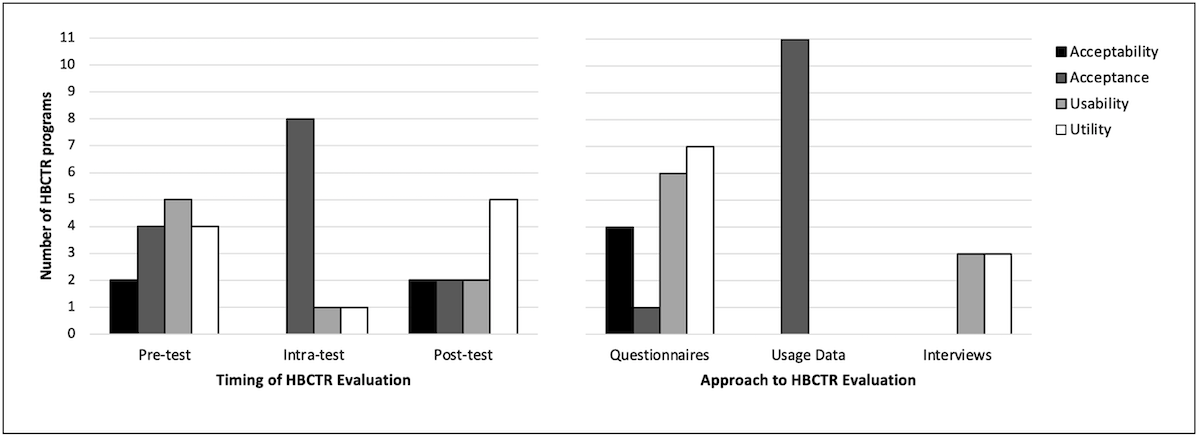
Evaluation timing (left) and approach (right) over the dimensions of the technology acceptance model constructs. HBCTR: home-based cardiac telerehabilitation.

Home-based cardiac telerehabilitation programs were commonly evaluated at the pretrial stage (n=5) and using a combination of intra and posttrial measures (n=4), followed by intratrial only (n=3), posttrial only (n=1) and a combination of pre-, intra-, and posttrial measures (n=1) (Figure 2). The following methods were used to evaluate home-based cardiac telerehabilitation: questionnaires (n=10); usage data (n=11); and interviews (n=3). Except for one study [22] that used the System Usability Scale, the remaining questionnaires used were ad hoc surveys. Yu et al [33] used a combination of captured usage data and patient-report questionnaires to evaluate acceptance of home-based cardiac telerehabilitation at both the intra- and posttrial stage. Higgins et al [26] used both questionnaires and interviews to evaluate both usability and utility of their home-based cardiac telerehabilitation program.

### Usability

Of the 18 studies, 7 studies [22,25-27,29,32,37] reported the usability of home-based cardiac telerehabilitation programs. Specific outcomes measures within the usability construct included perceived ease of system use and navigation [22,25,29,32], ease and comfort of use of wearable devices [27], system learnability [22,26], and comprehension and ease of undertaking tasks on the system [27,37]. Overall, studies reported high usability rating scores and qualitative feedback from participants regarding home-based cardiac telerehabilitation use.

### Utility

Specific outcomes measures within the utility construct included perceived usefulness in supporting behavior change [21,23,26,27,37], in managing psychological well-being [21,26], in controlling symptoms [21], in tracking goals and progress [21,25-27,37], in reducing outpatient visits [23], and of the overall home-based cardiac telerehabilitation system [23,29,33]. Utility of home-based cardiac telerehabilitation was generally favorably perceived, with the exception of 2 studies [29,33] in which perceived usefulness of the system was rated poorly.

### Acceptability

High rates of acceptability were reported in 3 studies [22,27,33], ranging from 81.3% to 88% of participants who agreed that they would continue to use the home-based cardiac telerehabilitation system regularly after they had completed the study intervention period. Prior to system use, one study [24] reported an acceptability rate of 59.3% (participants who were potentially willing to participate in a home-based cardiac telerehabilitation program).

### Acceptance

**Figure 3:**
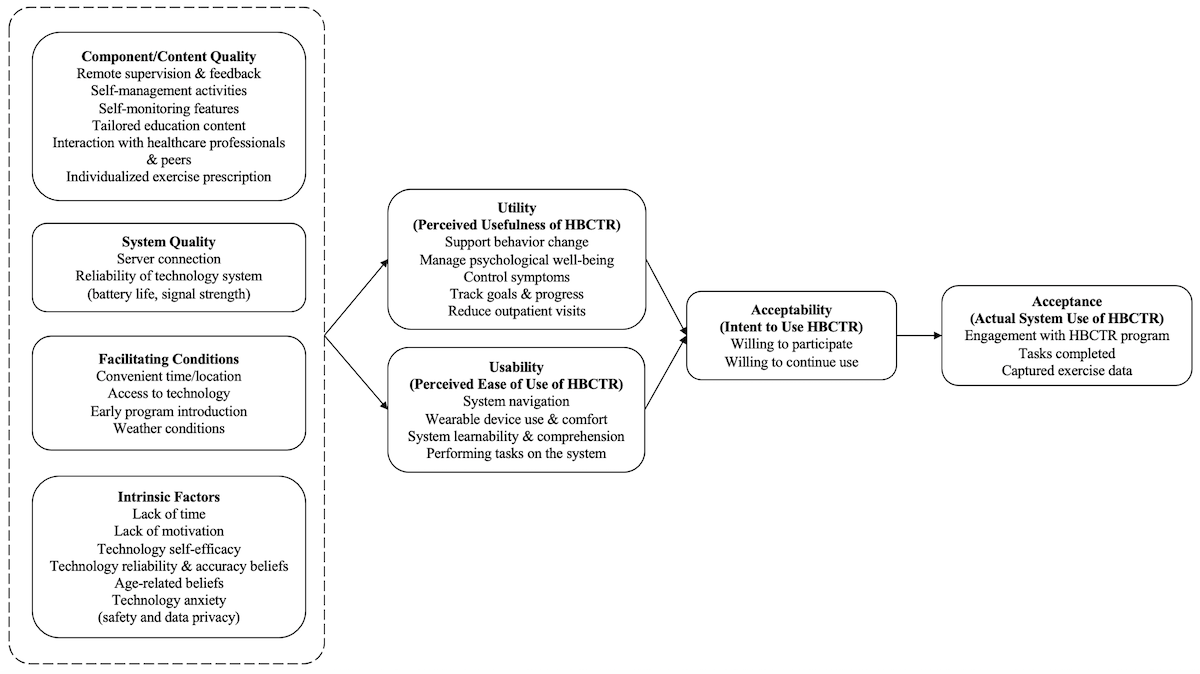
Technology acceptance of home-based cardiac telerehabilitation (HBCTR) programs.

Most studies reported participants’ usage of the home-based cardiac telerehabilitation system either through direct evaluation of program usage data or through self-reported participant survey responses (Table 1). Studies included a very broad range of outcome measures including engagement with home-based cardiac telerehabilitation [20,22,23,33,35,36] (ie, frequency and volume of website log-ins, smartphone app usage, activity tracker wear time); tasks completed [22,23,25,31,33] (ie, frequency and volume of educational modules reviewed, vitals logged, counseling sessions attended, response to program reminders), and captured exercise data [22,27,28,30,32,34] (ie, objective telemonitoring data on the uptake, adherence, and completion of prescribed exercise sessions and goals). Overall, usage was high, reflecting high end-user acceptance. Only 5 studies [22,25,30,33,34] reported usage data for specific components over time to determine the timepoints when participant usage tapered or ceased (Figure 3).

**Table 1:** Acceptance of home-based cardiac telerehabilitation programs.

Method, definition of actual use, and data timepoint				Acceptance of home-based cardiac telerehabilitation program
**Program usage data**				
	**Engagement with home-based cardiac telerehabilitation program**			
		6 weeks	Mean total number of 29 website log-ins (range 7-44; average 5 times per week) [20,21]	
		12 weeks	Mean total number of 50 website log-ins (range 26-86; average 4.2 times per week) [35] Wearable worn for a median of 61 of 84 study days (IQR 35-78) for a median of 12.7 hours (IQR 11.1-13.8) per day [22]; Mean decrease in wear time of 0.06 hours per week over 12 weeks [22]	
		16 weeks	Mean total number of 27 website log-ins (range 0-140) [36]	
		24 weeks	Proportion of participants who used and operated the app was 88.1% (4 weeks); 42.5% (8 weeks); 26.3% (12 weeks); 13.0% (16 weeks); 10.2% (20 weeks); 9.2% (24 weeks) [33]	
	**Tasks completed**			
		12 weeks	Participants completed an average off 66% (range 12.5%-100%) of weekly tasks (ie, intake form, heart rate upload, blood pressure data entry) [35] Median number of 11 weekly telephone counseling sessions attended; 91.7% of weekly telephone counseling sessions completed [22] Blood pressure recordings logged 3.6 (SD 2.1) times per week (at 4 weeks) and 3.6 (SD 1.9) (at 12 weeks); weight recordings logged 3.3 (SD 2.2) times per week (at 4 weeks) and 3.4 (SD 1.7) (at 12 weeks); mean 26.3 (SD 17.2) health-related messages text messages sent; reported exercises that met prespecified target heart rate an average of 3.5 (SD 1.4) times per week (at 4 weeks) and 3.5 (SD 1.1) times (at 12 weeks) [25]	
		16 weeks	41% of participants uploaded ≥32 exercise reports (average 2 exercise sessions per week); 26% of participants uploaded the required 8 blood pressure reports throughout study [36] Total of 122 individual chat sessions (mean 3.6 per participant) with either nurse, dietician, or exercise specialist [36] Participants used an average of 2.4, 2.6, and 2.7 hours of nursing, dietitian, and exercise specialist time, respectively [36]	
		24 weeks	Proportion of participants who responded to medication reminders and health questionnaires was 34% (4 weeks); 21.2% (8 weeks); 14.2% (12 weeks); 11% (16 weeks); 8.3% (20 weeks); 7.7% (24 weeks) [33]	
		52 weeks	96.3% of participants read education papers 4 times per month; 98.8% of participants consulted with their health care managers 1-4 times per month; 82.7% of participants sent their test results (ie, blood pressure and blood results) 4-8 times over 52 weeks [31]	
	**Captured exercise data**			
		6 weeks	86.6% of participants completed scheduled exercise sessions [32] Uptake^a^ rate: 80%; adherence^b^ rate: 94%; completion^c^ rate: 80% [30]	
		8 weeks	Uptake rate: 87%; adherence rate: 75%; completion^d^ rate: 75% [34]	
		12 weeks	86% of prescribed exercise goals completed over the 12-week study period; average decline of 8% completion per additional study week; 34% of walking goals completed over the 12-week study period; mean weekly increase in completion rate of 1% per additional week [22] Adherence rate to prescribed exercise was 58.34% (range 0-100) [27]	
		24 weeks	Participants exercised an average of 5.1 (SD 0.6) times a week; each time was 31.4 (SD 4.5) minutes [28]	
**Self-reported survey responses**				
	**Engagement with home-based cardiac telerehabilitation program**			
		24 weeks	100% of participants received WeChat modules and messages [23] 17.4% of participants reported using the app every day; 44.6% of participants often forgot to use the app [33]	
	**Tasks completed**			
		24 weeks	95% of participants read 75%-100% of WeChat modules and messages; 89% of participants read WeChat modules more than twice) [23]	

^a^Uptake was defined as attending baseline assessment, and uploading exercise data once to the home-based cardiac telerehabilitation platform.

^b^Adherence was defined as uploading 4 weeks of exercise data onto the home-based cardiac telerehabilitation.

^c^Completion was defined as attendance at the 6-week assessment.

^d^Completion was defined as attendance at the 8-week assessment.

### External Variables

#### Component Quality

The majority of the existing literature (n=8) on home-based cardiac telerehabilitation evaluation reported the program components that participants valued: remote supervision and feedback [21,24,27-29,37], support for self-management and self-monitoring [21,24,27,35,37], range of relevant educational modules [21,26,28], ability to communicate with health care professionals [21,27,35], and individualized exercise prescription [27]. Participants desired more interactive components such as chat platforms and noticeboards with peers to facilitate peer interaction and support [26,27,35] and greater intra- and postprogram support [27]. Participants in one study [26] wanted specific education content pertaining to death anxiety, and content that aligned rehabilitation goals with the purpose of living.

#### System Quality

Two studies [32,35] detailed participants’ perspectives on the technical efficiency of the home-based cardiac telerehabilitation system. Specifically, issues relating to server connection and reliability of the technology (ie, equipment battery life and signal strength) were reported as these influenced participants ability to engage with the program without interruption.

#### Intrinsic Factors

Participants reported several intrinsic factors at the individual level that influenced how they perceived home-based cardiac telerehabilitation programs (n=5). These included lack of time [21,27,37], lack of motivation [21,27,37], perceived self-efficacy in operating the telerehabilitation system [28], perceived reliability and accuracy of technology [24], apprehension related to safety and data privacy [24], and preconceived beliefs regarding the suitability of home-based cardiac telerehabilitation for older age [21,27].

#### Facilitating Conditions

The existence of resource and situational factors facilitated the usage of home-based cardiac telerehabilitation programs in included studies (n=5). Participants valued the accessibility and convenience offered by home-based cardiac telerehabilitation as it overcame restrictions related to time and location [21,27,37], but some expressed that regular access to the internet and computers would have facilitated uninterrupted usage of the program in earlier studies [29,37]. Situational factors such as timing of program introduction also influenced participants’ perception of home-based cardiac telerehabilitation usage [21,26]. Participants reported wanting the program to begin sooner after their diagnosis to facilitate early establishing of routines and prevent potential cardiac complications [21,26]. Wet and cold seasons were reported as a barrier to outdoor physical exercises [21].

Details of the external variables, usability, utility, and acceptability of home-based cardiac telerehabilitation reported in included studies can be found in Table S5 (Multimedia Appendix 2).

## Discussion

### Principal Findings

In our scoping review, we found that most evaluations were undertaken at the intratrial and posttrial stage using singular methodological approaches, and although home-based cardiac telerehabilitation had high usability, utility, acceptability, and acceptance, patients reported a number of external variables such as component quality, system quality, intrinsic factors, and facilitating conditions that influenced how they interacted with the home-based cardiac telerehabilitation program.

### Timing of Home-Based Cardiac Telerehabilitation Evaluation

Early evaluation of end-user acceptance and feasibility issues can critically inform the development and design of digital interventions and mitigate risks that an intervention is later undesirable or even abandoned at trial implementation stages [10,38]. Through our scoping review, we found that the majority of home-based cardiac telerehabilitation programs reported evaluations of technology acceptance either during or after trial implementation; evaluations were rarely reported at the pretrial stage. This may reflect a tendency in implementation research to prioritize the evaluation of trial intervention effectiveness over trial implementation effectiveness [39]. Yet, achieving intended trial effects is greatly dependent on participants’ sufficient engagement with the implemented technology in a trial that strongly appeals to their contextual health care needs [10]. Hence, there is a need for future research on home-based cardiac telerehabilitation to refocus efforts of program evaluation more upstream, so that identified technology acceptance issues can be addressed and programs finetuned to ensure optimal success before trial implementation.

### Approaches to Home-Based Cardiac Telerehabilitation Evaluation

Our review of the methodological approaches used to evaluate the technology acceptance constructs in home-based cardiac telerehabilitation revealed 3 main concerns. First, although home-based cardiac telerehabilitation programs used either quantitative (ie, survey questionnaires) or qualitative (ie, interviews) approaches to evaluate usability, utility, and acceptability, only 3 studies [21,26,37] employed qualitative methods (Figure 2), and only one study [26] used both approaches in tandem to evaluate the same technology acceptance attribute. Questionnaires are usually inexpensive and useful in gathering quantitative data in large samples but lack the ability to facilitate comprehension of in-depth individual variation in behaviors, perspectives, and experiences that qualitative interviews provide [40]. Such information is crucial to designing and delivering home-based cardiac telerehabilitation programs that truly match patients’ needs and preferences. We recommend that future home-based cardiac telerehabilitation programs employ a mixed methods approach, comprising both quantitative and qualitative methods to guarantee evaluation results that are practical, interpretable, and comprehensive [40].

Second, apart from one study [22] that used the System Usability Scale questionnaire, the remaining home-based cardiac telerehabilitation programs in this review used customized ad hoc questionnaires to measure the constructs of technology acceptance. This corresponds with the findings of previous reviews [38,41], which mostly included studies that evaluated digital health technology acceptance attributes using quantitative measures that lacked the psychometric properties of reliability and validity. This finding highlights an apparent scarcity of validated tools to evaluate technology acceptance in the context of digital health [38]. Furthermore, this could reflect the need for researchers to develop their own questionnaires that consider program-specific components, with general acceptance concepts, to allow for an assessment of technology acceptance attributes that is tailored to the particular home-based cardiac telerehabilitation context and population. However, this makes comparing results across studies challenging. It would be commendable to see future research efforts dedicated to adapting existing questionnaires or even validating new tools that encompass the unique home-based cardiac telerehabilitation context. We believe that having such generalizable measures can greatly advance home-based cardiac telerehabilitation research and practice by creating opportunities for comparable data on technology acceptance constructs to be analyzed and for comparative benchmarks to be set in home-based cardiac telerehabilitation program evaluation.

Third, home-based cardiac telerehabilitation programs had varied definitions and measurements of acceptance (ie, actual system usage) (Table 1). This is consistent with previous literature on the use of digital health technologies for cardiovascular disease self-management [42] and may be indicative of attempts to examine the multifarious behavior changes addressed in cardiac rehabilitation. Given that user engagement with technology is a dynamic process occurring in a self-directed manner by which users continually decide to either use or abandon a technology system [38,43], evaluations of home-based cardiac telerehabilitation acceptance should account for this temporal nature and analyze how usage evolves over the course of the rehabilitation program. This is especially important as interventions such as home-based cardiac telerehabilitation are theorized to require sustained use over time to realize intended effects. However, only 5 home-based cardiac telerehabilitation programs [22,25,30,33,34] reported usage over time (date-tagged acceptance data). Gallagher and Zhang [10] recommend the clear identification of individual digital health components targeted at behavior change and the integration of software capabilities that can monitor the usage of respective components. As the eventual goal of home-based cardiac telerehabilitation programs is successful incorporation into clinical practice, it would be interesting to see future studies examine the causal relationships between the level of home-based cardiac telerehabilitation usage and objective intervention outcome over time to determine the specific dose of a home-based cardiac telerehabilitation component needed to achieve optimal behavioral, physiological, and clinical outcomes.

### Technology Acceptance of Home-Based Cardiac Telerehabilitation

The acceptance rates observed in our review could be explained by the high usability, utility, and acceptability reported in the programs and correspond to the fundamental basis of the technology acceptance model, that is, that technology acceptance is determined by the degree of value and perceived burden [11]. This finding not only offers validation to the technology acceptance model but points to the potential of home-based cardiac telerehabilitation to revolutionize the landscape of secondary prevention by blending traditional services provided by health care professionals with technology-enabled self-care platforms to continue the provision of patient-centered care. This is especially crucial during the current COVID-19 pandemic to mitigate the demand for in-person services [4]. The suitability of home-based cardiac telerehabilitation as an effective alternative to center-based cardiac rehabilitation has been recently reported [7], with prospects for significant economic cost-savings through improved productivity and health outcomes [44]. Yet, an evaluation of end-user acceptance is foundational if barriers and gaps to patient uptake are to be addressed, and if successful wide-scale implementation of home-based cardiac telerehabilitation into clinical practice is to be realized. In the context of home-based cardiac telerehabilitation for patients with coronary heart disease, our review underlined the external variables that have influenced patient’s perceived usability and utility of home-based cardiac telerehabilitation. Recommendations for addressing these variables are offered in in the following paragraphs and may serve to provide a foundation for the development and design of future home-based cardiac telerehabilitation programs (Table 2).

**Table 2:** Recommendations to improve home-based cardiac telerehabilitation acceptance and its evaluation.

Topic	Recommendation
Evaluation timing	Home-based cardiac telerehabilitation program evaluation should be undertaken throughout the entirety of the developmental and implementation, ie, before, during and after trial implementation.
Evaluation approach	Home-based cardiac telerehabilitation program evaluation should employ a mixed approach comprising of both quantitative and qualitative methods. Measurement tools must be tailored to encompass the unique context of home-based cardiac telerehabilitation by adapting existing questionnaires or validating new ones. Evaluations of home-based cardiac telerehabilitation technology acceptance should analyze how usage of individual program components evolves over the course of the rehabilitation program. Causal relationships between home-based cardiac telerehabilitation usage and intervention outcomes should be examined to determine specific doses needed to achieve optimal behavioral, physiological, and clinical outcomes.
Design and testing	Developers should prioritize user-centered approaches by partnering with end users (ie, clinicians and patients) in the co-designing of programs in the early stages of program design. Field-testing and evaluations of the technologies supporting home-based cardiac telerehabilitation services should occur prior to trial implementation stages.
Individualization	Home-based cardiac telerehabilitation programs should be offered as early as possible for patients. Alternatives for either indoor or outdoor exercise training should be programmed.
Accessibility	Home-based cardiac telerehabilitation programs should be adapted to the socioeconomic needs of end users and their community Partnerships with local governing bodies should be established to marshal resources and secure funding to invest in required infrastructure. The prospects of insurance coverage for home-based cardiac telerehabilitation programs should be explored. home-based cardiac telerehabilitation programs should be reasonably priced with subsidies for mobile phones, data plans and wearables.
Data privacy and security	Home-based cardiac telerehabilitation should provide patients with transparent privacy policies and comply with data governance regulations and security protocols.
Training	Patients should be provided introductory training sessions that are supported by practical step-by-step instruction manuals.
Technology support	Designated technical support staff should be made available on home-based cardiac telerehabilitation platforms.

### Recommendations for Home-Based Cardiac Telerehabilitation Development

Home-based cardiac telerehabilitation developers should prioritize user-centered approaches by partnering with end users (ie, clinicians and patients) in the co-design, field test, and evaluation of technologies supporting telerehabilitation services [10]. Accounting for the needs and preferences of patients in the early stages of program design can help mitigate concerns regarding home-based cardiac telerehabilitation component quality and can help in identifying issues with home-based cardiac telerehabilitation system quality program testing prior to trial implementation stages. However, we observed that less than one-fifth of home-based cardiac telerehabilitation programs reported including end users in the design and development stage and just over half undertook user testing of the home-based cardiac telerehabilitation system (Table S4 in Multimedia Appendix 2). American Heart Association’s recommendations on home-based cardiac telerehabilitation [6] and the Beatty et al [45] framework for mobile technology in cardiac rehabilitation can guide the development and evaluation of future home-based cardiac telerehabilitation programs.

Facilitating conditions, such as the timing of program introduction, prevailing weather conditions, and access to internet and computers, were reported to influence patients’ use of home-based cardiac telerehabilitation programs. Given that peak lifestyle changes occur in the first 6 months after diagnosis [46] and that early cardiac rehabilitation is a significant predictor of cardiac function and functional capacity [47,48], home-based cardiac telerehabilitation should be offered as early as possible for patients to ensure optimal outcomes. Home-based cardiac telerehabilitation should also offer patients alternatives for either indoor or outdoor exercise training, especially in regions with seasonal weather changes. Additionally, as inequities in cardiovascular health still exist, an examination of socioeconomic characteristics are crucial if technology accessibility and affordability issues surrounding home-based cardiac telerehabilitation usage are to be addressed [49,50]. Access to technology infrastructure remains unevenly distributed worldwide, with internet use being significantly lower in low- and middle-income regions than in high-income regions [51]. Partnerships with local governing bodies should be established to marshal resources and secure funding to invest in required infrastructure [52]. Collaborating with nongovernment organizations to advocate for prospects on insurance coverage and to negotiate reasonable pricing and subsidies for mobile phones, data plans, and wearables will aid in supporting the long-term implementation and scale-up of home-based cardiac telerehabilitation in clinical practice [53].

Although intrinsic factors such as lack of time and motivation are less amenable to change, program adaptations can be made to palliate concerns regarding data privacy, perceived technology self-efficacy and reliability, and preconceived age-related beliefs regarding home-based cardiac telerehabilitation usage. Program training, technological support, and the availability of transparent privacy policies, especially for older adults, can reduce potential uneasiness and facilitate willingness to engage in digital health technologies such as home-based cardiac telerehabilitation [4,54,55]. Even though the majority of included home-based cardiac telerehabilitation provided face-to-face program training, less than one-third offered ongoing technological support during program intervention, and only half indicated using secure password-protected platforms (Table S4 in Multimedia Appendix 2). Future programs should develop introductory training sessions that are supported by practical step-by-step instruction manuals with designated technical support staff on home-based cardiac telerehabilitation platforms that comply with data governance regulations and security protocols to mitigate the risk of privacy breaches [54,55].

### Limitations

This scoping review has some limitations that need to be acknowledged. First, the inclusion of only English-language papers may have resulted in the omission of eligible papers published in other languages. However, our comprehensive search strategy and broad inclusion of different study designs with no time restrictions allows for breadth and depth of inclusion in this review. Second, although the technology acceptance model offers a user-centered approach in mapping patient perspectives of home-based cardiac telerehabilitation program acceptance, content analysis is inherently reductive and could have limited the scope of our findings. However, the thematic analysis undertaken to explore the external variables influencing home-based cardiac telerehabilitation acceptance could have mitigated the risks of missing meaningful data from the studies included in our review. Lastly, although end users in this user-centered approach also include health care providers delivering home-based cardiac telerehabilitation, the evaluation of technology acceptance from provider perspectives was not included because it was not the focus of this review. It is likely that the underlying determinants of home-based cardiac telerehabilitation acceptance may differ in these users. We recommend that future research in the field of home-based cardiac telerehabilitation aim to include literature in other languages, utilize other available conceptual frameworks on digital health acceptance, and accommodate perspectives from different categories of end users in order to fully comprehend and address home-based cardiac telerehabilitation implementation and acceptance.

### Conclusions

We drew on the technology acceptance model to map available research on patient’s technology acceptance of home-based cardiac telerehabilitation. Our results demonstrated that, while patient perspectives on home-based cardiac telerehabilitation usability, utility, acceptability, and acceptance were high, a number of external variables influence technology acceptance of home-based cardiac telerehabilitation programs. Additionally, gaps in current home-based cardiac telerehabilitation evaluation timing and approaches were revealed. As the appeal for home-based cardiac telerehabilitation grows during the COVID-19 pandemic and beyond, findings from this review can be used to provide guidance for stakeholders and clinicians in developing and evaluating patient-centered home-based cardiac telerehabilitation programs.

## Appendix

Multimedia Appendix 1 Search strategy.

Multimedia Appendix 2 Summary, characteristics, and outcomes of studies.
