# Supplementary material for: Technology Acceptance of Home-Based Cardiac Telerehabilitation Programs in Patients With Coronary Heart Disease: Systematic Scoping Review
Source: J Med Internet Res. 2022 Jan 7;24(1):e34657. doi: 10.2196/34657 (PMC8783276; doi:10.2196/34657)
Supplement: Multimedia Appendix 1 [file jmir_v24i1e34657_app1.pdf]

Multimedia Appendix 1. Search Strategy

Table S1. Index Terms and Keywords for searching in seven databases
Search stopped on 7 July 2021

Database: PubMed

Search No.	Indexed and Keyword Terms
1	"Myocardial Ischemia"[Mesh] OR "Coronary Artery Disease"[Mesh] OR "Acute Coronary Syndrome"[Mesh] OR "Myocardial Infarction"[Mesh] OR "Angina Pectoris"[Mesh] OR "Percutaneous Coronary Intervention"[Mesh] OR "Coronary Artery Bypass"[Mesh]
2	Myocardial Ischemia[Title/Abstract] OR Ischemic Heart Disease[Title/Abstract] OR Heart Disease*[Title/Abstract] OR Heart attack[Title/Abstract] OR Coronary Heart Disease[Title/Abstract] OR Coronary Artery Disease[Title/Abstract] OR Coronary Disease*[Title/Abstract] OR Acute Coronary Syndrome[Title/Abstract] OR Acute Coronary Syndrom*[Title/Abstract] OR Myocardial Infarction[Title/Abstract] OR Angina*[Title/Abstract] OR Percutaneous Coronary Intervention[Title/Abstract] OR Percutaneous Coronary Revascularization*[Title/Abstract] OR Percutaneous Transluminal Coronary Angioplast*[Title/Abstract] OR Coronary Balloon Angioplast*[Title/Abstract] OR Coronary Artery Bypass*[Title/Abstract]
3	#1 OR #2
4	("Rehabilitation"[Mesh]) OR (Rehabilitation[Title/Abstract] OR rehab[Title/Abstract])
5	"Telemedicine"[Mesh] OR mhealth[Title/Abstract] OR m-health[Title/Abstract] OR mobile health[Title/Abstract] OR mobile application[Title/Abstract] OR mobile device[Title/Abstract] OR mobile communication*[Title/Abstract] OR mobile phone[Title/Abstract] OR smartphone[Title/Abstract] OR smart phone[Title/Abstract] OR smartphone application[Title/Abstract] OR cellphone[Title/Abstract] OR cellular phone[Title/Abstract] OR telemedicine[Title/Abstract] OR telerehabilitation[Title/Abstract] OR tele-rehabilitation[Title/Abstract] OR Virtual Rehabilitation[Title/Abstract] OR Remote Rehabilitation[Title/Abstract] OR telehealth[Title/Abstract] OR tele-health[Title/Abstract] OR telemonitor[Title/Abstract] OR ehealth[Title/Abstract] OR e-health[Title/Abstract] OR digital health[Title/Abstract] OR mobile technolog*[Title/Abstract] OR website[Title/Abstract] OR web-based[Title/Abstract]
6	#3 AND #4 AND #5

This is a Multimedia Appendix to a full manuscript published in the J Med Internet Res. For full copyright and citation information see https://www.jmir.org/2022/1/e34657

Database: Cochrane Library (CENTRAL)

Search No.	Indexed and Keyword Terms
1	MeSH descriptor: [Myocardial Ischemia] explode all trees
2	MeSH descriptor: [Coronary Artery Disease] explode all trees
3	MeSH descriptor: [Acute Coronary Syndrome] explode all trees
4	MeSH descriptor: [Myocardial Infarction] explode all trees
5	MeSH descriptor: [Angina Pectoris] explode all trees
6	MeSH descriptor: [Percutaneous Coronary Intervention] explode all trees
7	MeSH descriptor: [Coronary Artery Bypass] explode all trees
8	'myocardial ischemia':ab,ti OR 'ischemic heart disease':ab,ti OR 'heart disease*':ab,ti OR 'heart attack':ab,ti OR 'coronary heart disease':ab,ti OR 'coronary artery disease':ab,ti OR 'coronary disease*':ab,ti OR 'acute coronary syndrome':ab,ti OR 'acute coronary syndrom*':ab,ti OR 'myocardial infarction':ab,ti OR 'angina*':ab,ti OR 'percutaneous coronary intervention':ab,ti OR 'percutaneous coronary revascularization*':ab,ti OR 'percutaneous transluminal coronary angioplast*':ab,ti OR 'coronary balloon angioplast*':ab,ti OR 'coronary artery bypass*':ab,ti
9	#1 OR #2 OR #3 OR #4 OR #5 OR #6 OR #7 OR #8
10	MeSH descriptor: [Rehabilitation] explode all trees
11	'rehabilitation':ab,ti OR 'rehab':ab,ti
12	#10 OR #11
13	MeSH descriptor: [Telemedicine] explode all trees
14	'telemedicine':ab,ti OR 'mhealth':ab,ti OR 'm-health':ab,ti OR 'mobile health':ab,ti OR 'mobile application':ab,ti OR 'mobile device':ab,ti OR 'mobile communication*':ab,ti OR 'mobile phone':ab,ti OR 'smartphone':ab,ti OR 'smart phone':ab,ti OR 'smartphone application':ab,ti OR 'cellphone':ab,ti OR 'cellular phone':ab,ti OR 'telemedicine':ab,ti OR 'telerehabilitation':ab,ti OR 'tele-rehabilitation':ab,ti OR 'virtual rehabilitation':ab,ti OR 'remote rehabilitation':ab,ti OR 'telehealth':ab,ti OR 'tele-health':ab,ti OR 'telemonitor':ab,ti OR 'ehealth':ab,ti OR 'e-health':ab,ti OR 'digital health':ab,ti OR 'mobile technolog*':ab,ti OR 'website':ab,ti OR 'web-based':ab,ti
15	#13 OR #14
16	#9 AND #12 AND #15

This is a Multimedia Appendix to a full manuscript published in the J Med Internet Res. For full copyright and citation information see https://www.jmir.org/2022/1/e34657

Database: Embase

Search No.	Indexed and Keyword Terms
1	'heart muscle ischemia'/exp OR 'coronary artery disease'/exp OR 'acute coronary syndrome'/exp OR 'heart infarction'/exp OR 'angina pectoris'/exp OR 'percutaneous coronary intervention'/exp OR 'coronary artery bypass graft'/exp
2	'myocardial ischemia':ab,ti OR 'ischemic heart disease':ab,ti OR 'heart disease*':ab,ti OR 'heart attack':ab,ti OR 'coronary heart disease':ab,ti OR 'coronary artery disease':ab,ti OR 'coronary disease*':ab,ti OR 'acute coronary syndrome':ab,ti OR 'acute coronary syndrom*':ab,ti OR 'myocardial infarction':ab,ti OR 'angina*':ab,ti OR 'percutaneous coronary intervention':ab,ti OR 'percutaneous coronary revascularization*':ab,ti OR 'percutaneous transluminal coronary angioplast*':ab,ti OR 'coronary balloon angioplast*':ab,ti OR 'coronary artery bypass*':ab,ti
3	#1 OR #2
4	'rehabilitation'/exp OR 'rehabilitation':ab,ti OR 'rehab':ab,ti
5	'telemedicine'/exp OR 'mhealth':ab,ti OR 'm-health':ab,ti OR 'mobile health':ab,ti OR 'mobile application':ab,ti OR 'mobile device':ab,ti OR 'mobile communication*':ab,ti OR 'mobile phone':ab,ti OR 'smartphone':ab,ti OR 'smart phone':ab,ti OR 'smartphone application':ab,ti OR 'cellphone':ab,ti OR 'cellular phone':ab,ti OR 'telemedicine':ab,ti OR 'telerehabilitation':ab,ti OR 'tele-rehabilitation':ab,ti OR 'virtual rehabilitation':ab,ti OR 'remote rehabilitation':ab,ti OR 'telehealth':ab,ti OR 'tele-health':ab,ti OR 'telemonitor':ab,ti OR 'ehealth':ab,ti OR 'e-health':ab,ti OR 'digital health':ab,ti OR 'mobile technolog*':ab,ti OR 'website':ab,ti OR 'web-based':ab,ti
6	#3 AND #4 AND #5

This is a Multimedia Appendix to a full manuscript published in the J Med Internet Res. For full copyright and citation information see https://www.jmir.org/2022/1/e34657

Database: CINAHL

Search No.	Indexed and Keyword Terms
1	(MH "Myocardial Ischemia+") OR (MH "Myocardial Infarction+") OR (MH "Coronary Artery Bypass+") OR (MH "Coronary Disease+") OR (MH "Coronary Arteriosclerosis") OR (MH "Coronary Arteriosclerosis") OR (MH "Acute Coronary Syndrome") OR (MH "Angina Pectoris+") OR (MH "Percutaneous Coronary Intervention") OR (MH "Angioplasty, Transluminal, Percutaneous Coronary") OR TI ('Myocardial Ischemia' OR 'Ischemic Heart Disease' OR 'Heart Disease*' OR 'Heart attack' OR 'Coronary Heart Disease' OR 'Coronary Artery Disease' OR 'Coronary Disease*' OR 'Acute Coronary Syndrome' OR 'Acute Coronary Syndrom*' OR 'Myocardial Infarction' OR 'Angina*' OR 'Percutaneous Coronary Intervention' OR 'Percutaneous Coronary Revascularization*' OR 'Percutaneous Transluminal Coronary Angioplast*' OR 'Coronary Balloon Angioplast*' OR 'Coronary Artery Bypass*') OR AB ('Myocardial Ischemia' OR 'Ischemic Heart Disease' OR 'Heart Disease*' OR 'Heart attack' OR 'Coronary Heart Disease' OR 'Coronary Artery Disease' OR 'Coronary Disease*' OR 'Acute Coronary Syndrome' OR 'Acute Coronary Syndrom*' OR 'Myocardial Infarction' OR 'Angina*' OR 'Percutaneous Coronary Intervention' OR 'Percutaneous Coronary Revascularization*' OR 'Percutaneous Transluminal Coronary Angioplast*' OR 'Coronary Balloon Angioplast*' OR 'Coronary Artery Bypass*')
2	(MH "Rehabilitation+") OR TI ('rehabilitation' OR 'rehab') OR AB ('rehabilitation' OR 'rehab')
3	(MH "Telemedicine+") OR (MH "Telehealth+") OR TI ('mhealth' OR 'm-health' OR 'mobile health' OR 'mobile application' OR 'mobile device' OR 'mobile communication*' OR 'mobile phone' OR 'smartphone' OR 'smart phone' OR 'smartphone application' OR 'cellphone' OR 'cellular phone' OR 'telemedicine' OR 'telerehabilitation' OR 'tele-rehabilitation' OR 'Virtual Rehabilitation' OR 'Remote Rehabilitation' OR 'telehealth' OR 'tele-health' OR 'telemonitor' OR 'ehealth' OR 'e-health' OR 'digital health' OR 'mobile technolog*' OR 'website' OR 'web-based') OR AB ('mhealth' OR 'm-health' OR 'mobile health' OR 'mobile application' OR 'mobile device' OR 'mobile communication*' OR 'mobile phone' OR 'smartphone' OR 'smart phone' OR 'smartphone application' OR 'cellphone' OR 'cellular phone' OR 'telemedicine' OR 'telerehabilitation' OR 'tele-rehabilitation' OR 'Virtual Rehabilitation' OR 'Remote Rehabilitation' OR 'telehealth' OR 'tele-health' OR 'telemonitor' OR 'ehealth' OR 'e-health' OR 'digital health' OR 'mobile technolog*' OR 'website' OR 'web-based')
4	#1 AND #2 AND #3

This is a Multimedia Appendix to a full manuscript published in the J Med Internet Res. For full copyright and citation information see https://www.jmir.org/2022/1/e34657

Database: PsycINFO

Search No.	Indexed and Keyword Terms
1	exp Cardiovascular Disorders/ OR exp Myocardial Infarctions/
2	(Myocardial Ischemia OR Ischemic Heart Disease OR Heart Disease* OR Heart Attack OR Coronary Heart Disease OR Coronary Artery Disease OR Coronary Disease* OR Acute Coronary Syndrome OR Acute Coronary Syndrome* OR Myocardial Infarction OR Angina* OR Percutaneous Coronary Intervention OR Percutaneous Coronary Revascularisation* OR Percutaneous Transluminal Coronary Angioplast* OR Coronary Balloon Angioplast* OR Coronary Artery Bypass*).ti,ab.
3	#1 OR #2
4	exp Rehabilitation/
5	(Rehabilitation or rehab).ti,ab.
6	#4 OR #5
7	exp Telemedicine/
8	(mhealth OR m-health OR mobile health OR mobile application OR mobile device OR mobile communication* OR mobile phone OR smartphone OR smart phone OR smartphone application OR cellphone OR cellular phone OR telemedicine OR telerehabilitation OR tele-rehabilitation OR Virtual Rehabilitation OR Remote Rehabilitation OR telehealth OR tele-health OR telemonitor OR ehealth OR e-health OR digital health OR mobile techolog* OR website OR web-based).ti,ab.
9	#7 OR #8
10	#3 AND #6 AND #9

This is a Multimedia Appendix to a full manuscript published in the J Med Internet Res. For full copyright and citation information see https://www.jmir.org/2022/1/e34657

Database: Scopus

Search No.	Indexed and Keyword Terms
1	TITLE-ABS-KEY(("Myocardial Ischemia" OR "Ischemic Heart Disease" OR "Heart Disease*" OR "Heart attack" OR "Coronary Heart Disease" OR "Coronary Artery Disease" OR "Coronary Disease*" OR "Acute Coronary Syndrome" OR "Acute Coronary Syndrom*" OR "Myocardial Infarction" OR "Angina*" OR "Percutaneous Coronary Intervention" OR "Percutaneous Coronary Revascularization*" OR "Percutaneous Transluminal Coronary Angioplast*" OR "Coronary Balloon Angioplast*" OR "Coronary Artery Bypass*") AND ("rehabilitation" OR "rehab") AND ("mhealth" OR "m-health" OR "mobile health" OR "mobile application" OR "mobile device" OR "mobile communication*" OR "mobile phone" OR "smartphone" OR "smart phone" OR "smartphone application" OR "cellphone" OR "cellular phone" OR "telemedicine" OR "telerehabilitation" OR "tele-rehabilitation" OR "Virtual Rehabilitation" OR "Remote Rehabilitation" OR "telehealth" OR "tele-health" OR "telemonitor" OR "ehealth" OR "e-health" OR "digital health" OR "mobile technolog*" OR "website" OR "web-based"))

Table S2. List of Excluded Studies

Author (Year)	Title	Reason
Batalik et al. (2021)	Cardiac Rehabilitation Based on the Walking Test and Telerehabilitation Improved Cardiorespiratory Fitness in People Diagnosed with Coronary Heart Disease during the COVID-19 Pandemic.	Irrelevant/Mixed Intervention: Teleconsultations only
Beatty et al. (2013)	Using mobile technology for cardiac rehabilitation: a review and framework for development and evaluation.	Inappropriate study design: Review
Beatty et al. (2018)	VA FitHeart, a Mobile App for Cardiac Rehabilitation: Usability Study	Inappropriate participants: HF patients
Busnatu et al. (2015)	FI-STAR online personalized cardiac rehabilitation solution.	Irrelevant outcomes
Busnatu et al. (2016)	Quality of experience evaluation of an online cardiac rehabilitation mhealth solution	Conference Abstract only
Chen et al. (2011)	Food for the Heart: Understanding Responses of Patients Enrolled in Cardiac Rehabilitation Toward Web-Based and Mobile-Based Nutrition Tools	Inappropriate participants: included caregivers
da Cruz et al. (2021)	Randomized Controlled Crossover Trial of Virtual Reality in Maintenance Cardiovascular Rehabilitation in a Low-Resource Setting: impact on Adherence, Motivation, and Engagement.	Inappropriate participants: HF patients
Dale et al. (2015)	Acceptability of a Mobile Health Exercise-Based Cardiac Rehabilitation Intervention	Irrelevant/Mixed Intervention: hybrid intervention – included CBCR
Deighan et al. (2017)	The Digital Heart Manual: A pilot study of an innovative cardiac rehabilitation programme developed for and with users	Inappropriate participants: included spouses and healthcare practitioners
Dithmer et al. (2016)	The Heart Game": Using Gamification as Part of a Telerehabilitation Program for Heart Patients.	Inappropriate participants: HF patients
Eckardt et al. (2021)	Smartphone-guided secondary prevention for patients with coronary artery disease	Inappropriate participants: patients with risk factors only included
Ensom et al. (2019)	Feasibility of, and adherence to, a novel, home-based cardiac tele-rehabilitation program for heart attack survivors: The mi-pace study.	Abstract only

This is a Multimedia Appendix to a full manuscript published in the J Med Internet Res. For full copyright and citation information see https://www.jmir.org/2022/1/e34657

Forman et al. (2014)	Utility and Efficacy of a Smartphone Application to Enhance the Learning and Behavior Goals of Traditional Cardiac Rehabilitation	Irrelevant/Mixed Intervention: hybrid intervention – included CBCR
Freene et al. (2020)	Behavioral Change Smartphone App and Program (ToDo-CR) to Decrease Sedentary Behavior in Cardiac Rehabilitation Participants: Prospective Feasibility Cohort Study.	Irrelevant/Mixed Intervention: hybrid intervention – included CBCR
Gallagher et al. (2016)	Technology use and confidence in cardiac rehabilitation patients.	Irrelevant outcomes
Gallagher et al. (2017)	Feasibility of smartphone app use for secondary prevention in cardiac patients.	Abstract only
Harris et al. (2003)	Cardiac rehabilitation with nurse care management and telephonic interactions at a community hospital: program evaluation of participation and lipid outcomes.	Irrelevant outcomes
Houchen-Wolloff et al. (2018)	A web-based cardiac rehabilitation alternative for those declining or dropping out of conventional rehabilitation	Irrelevant outcomes
Jameie et al. (2019)	Development and Usability Evaluation of Web-Based Telerehabilitation Platform for Patients After Myocardial Infarction.	Irrelevant outcomes
Kerr et al. (2010)	The Potential of Web-based Interventions for Heart Disease Self-Management: A Mixed Methods Investigation	Irrelevant/Mixed Intervention: not CR
Kerr et al. (2008)	Applying user-generated quality criteria to develop an Internet intervention for patients with heart disease	Irrelevant/Mixed Intervention: not CR
Lin et al. (2018)	A pilot-study to assess the feasibility and acceptability of an Internet-based cognitive-behavior group therapy using video conference for patients with coronary artery heart disease.	Irrelevant/Mixed Intervention: not CR
Melholt et al. (2017)	Cardiac patients' experiences with a telerehabilitation web portal: Implications for eHealth literacy.	Irrelevant outcomes
Nabutovsky et al. (I2020)	Feasibility, Safety, and Effectiveness of a Mobile Application in Cardiac Rehabilitation	Irrelevant outcomes
O'Shea et al. (2020)	A qualitative exploration of cardiovascular disease patients' views and experiences with an eHealth cardiac rehabilitation intervention: The PATHway Project	Irrelevant/Mixed Intervention: Phase 3 CBCR
Piotrowicz et al. (2014)	Feasibility of home-based cardiac telerehabilitation: Results of teleintermed study.	Inappropriate participants: HF patients

This is a Multimedia Appendix to a full manuscript published in the J Med Internet Res. For full copyright and citation information see https://www.jmir.org/2022/1/e34657

Salvi et al., 2018	An m-Health system for education and motivation in cardiac rehabilitation: the experience of HeartCycle guided exercise.	Irrelevant/Mixed Intervention: Phase 3 CBCR
Sankaran et al. (2016)	A Grounded Approach for Applying Behavior Change Techniques in Mobile Cardiac Tele-Rehabilitation	Inappropriate participants: HF patients
Sankaran et al. (2019)	Evaluating the Impact of the HeartHab App on Motivation, Physical Activity, Quality of Life, and Risk Factors of Coronary Artery Disease Patients: Multidisciplinary Crossover Study	Irrelevant outcomes
Scherrenberg et al. (2021)	Patient experiences and willingness-to-pay for cardiac telerehabilitation during the first surge of the COVID-19 pandemic: single-centre experience	Irrelevant/Mixed Intervention: hybrid intervention – included CBCR
Sengupta et al. 2020	A Mobile Health Intervention System for Women With Coronary Heart Disease: Usability Study.	Inappropriate participants: HF patients
Spaulding et al. (2019)	Corrie Health Digital Platform for Self-Management in Secondary Prevention After Acute Myocardial Infarction.	Irrelevant outcomes
Spencer (2017)	Heart Online website: a physiotherapist's perspective.	Inappropriate participants: physiotherapists
Stamm-Balderjahn et al. (2019)	Development and evaluation of a patient passport to promote self-management in patients with heart diseases.	Irrelevant/Mixed Intervention: not CR
Walsh et al. (2018)	Electronic health physical activity behavior change intervention to self-manage cardiovascular disease: Qualitative exploration of patient and health professional requirements.	Inappropriate participants

This is a Multimedia Appendix to a full manuscript published in the J Med Internet Res. For full copyright and citation information see https://www.jmir.org/2022/1/e34657
