# Supplementary material for: Technology Acceptance of Home-Based Cardiac Telerehabilitation Programs in Patients With Coronary Heart Disease: Systematic Scoping Review
Source: J Med Internet Res. 2022 Jan 7;24(1):e34657. doi: 10.2196/34657 (PMC8783276; doi:10.2196/34657)
Supplement: Multimedia Appendix 2 [file jmir_v24i1e34657_app2.pdf]

Multimedia Appendix 2. Summary, Characteristics, and Outcomes of Studies

Table S3. Characteristics of Included Studies

Author. (Year)/ Country	Study<br>a. Design<br>b. Aim	Population:<br>a. Number of participants (*N*)<br>b. Diagnosis<br>c. Age (Mean ± SD)<br>d. Gender (female%)	Intervention<br>a. HBCTR programme<br>b. Duration	Method of Data Collection	Timing of Evaluation
Devi et al. (2014)/ United Kingdom	a. RCT<br>b. Examine the effectiveness of a Web-based CR program for those with angina	a. N = 95<br>b. Stable angina<br>c. 66.27 ± 8.35<br>d. 25.5%	a. Education on CHD and related RFs on secure "ActivateYourHeart" website. Individualized behavior goals modified depending on progress. Online exercise diary for tracking of daily PA via Sensewear Pro3 accelerometer. Contact with CR nurses via an online email link or at weekly scheduled synchronized chat rooms.<br>b. 6 weeks	Quantitative (captured usage data)	Intra-trial
Devi et al. (2014)/ United Kingdom	a. Descriptive qualitative<br>b. Explore patients' views on acceptability and feasibility of a new web-based CR programme.	a. *N* = 16<br>b. Stable angina, PCI or CABG<br>c. 66<br>d. 25%		Qualitative (semi-structured interviews)	Post-trial
Ding et al. (2021)/ United States of America	a. Pilot Usability study<br>b. Assess the feasibility of a cardiac telerehabilitation program to AMI survivors who declined center-based CR	a. *N* = 18<br>b. AMI<br>c. 59 ± 7<br>d. 33%	a. The MI-PACE tele-CR program included a validated wearable device connected via Bluetooth to an Android tablet app that displayed goals and progress for exercise. Counseling and education sessions with the nurse were scheduled weekly over the 12-week study period.<br>b. 12 weeks	Quantitative (captured usage data & SUS questionnaire)	Pre-trial
Dorje et al. (2019)/ China	a. RCT<br>b. Assess the acceptability and perceived utility of SMART-CR/SP	a. *N* = 125<br>b. Post-PCI<br>c. NR<br>d. NR	a. SMART-CR/SP app delivered weekly educational modules and motivational messages. Wireless data transfer of pedometer, BP and HR monitor to review weekly progress on secure data portal. Individualized support for RF management delivered by a cardiologist via WeChat-based consultations as required.<br>b. 24 weeks	Quantitative (survey questionnaire)	Post-trial
Fang et al. (2016)/ China	a. Cross-sectional<br>b. Assess attitudes towards acceptance of HBCTR technology	a. *N* = 150<br>b. Post-PCI<br>c. 63.3 ± 9.63<br>d. 22.7%	a. Customized exercise prescription, CHD education materials and real-time PA monitoring via a belt-strap sensor, a smartphone application, servers and a web portal. Customized feedback on captured data sent to patients through SMS.<br>b. 6 weeks	Quantitative (survey questionnaire)	Pre-trial
Harzand et al. (2018)/ United States of America	a. Feasibility study<br>b. Evaluate the feasibility and acceptability of a smartphone-enabled, home-based CR among veterans with CHD.	a. *N* = 18<br>b. ACS, AMI, post-PCI or CABG<br>c. 65 ± 5<br>d. 0%	a. The app featured daily exercise reminders, a virtual diary to document exercise sessions (type, duration, peak HR achieved) and vital signs, videos on heart conditions and RF modification, and 2-way messaging with a cardiology physician assistant.<br>b. 12 weeks	Quantitative (captured usage data & survey)	Pre-trial
Higgins et al. (2017)/ Australia	a. Pilot study<br>b. Determine patients' perceptions of the acceptability and utility of Help Yourself Online	a. *N* = 21<br>b. ACS<br>c. 62 ± 8<br>d. 19%	a. The Help Yourself Online program included modules on healthy eating, physical activity, medication adherence, smoking cessation, emotional management, and social support. Self-management activities included decisional balance activities, exploration of barriers/facilitators of behaviour change, action planning, & coping.<br>b. 2 to 3 weeks	Quantitative (survey questionnaire) & Qualitative (semi-structured focus	Pre-trial

This is a Multimedia Appendix to a full manuscript published in the J Med Internet Res. For full copyright and citation information see https://www.jmir.org/2022/1/e34657

	program after an acute cardiac event			group or telephone interviews)	
Rawstorn et al. (2018)/ New Zealand	a. Cross-sectional<br>b. Evaluate user experiences, usability, and acceptability, of REMOTE-CR.	a. *N* = 67<br>b. CHD (AMI, angina, CRV)<br>c. 61.3 (36 – 85)<br>d. 14.9%	a. REMOTE-CR comprised individualized exercise prescription and real-time monitoring/coaching, behavioral strategies, delivered via wearable sensor, mobile and web applications.<br>b. 12 weeks	Quantitative (captured usage data questionnaire & survey)	Combination of intra- & post-trial
Song et al. (2020)/ China	a. RCT<br>b. Evaluate the acceptance and compliance to smartphone-based telemonitored exercise rehabilitation among CHD patients	a. *N* = 48<br>b. Stable CHD<br>c. 54.2 ± 8.76<br>d. 10.4%	a. Telemonitoring smartphone software (MEMRS-CRS) and HR belts (Suunto) monitored HR during PA. Medical staff monitored patients' exercise frequency/intensity, BP and HR before and after exercise at computer terminal and communicated with patients weekly through text messaging and telephone call.<br>b. 24 weeks	Quantitative (captured usage data & survey)	Combination of intra- & post-trial
Varnfield et al. (2011)/ Australia	a. Cross-sectional<br>b. Evaluate the acceptability of and adherence to the CAP CR program	a. *N* = 15<br>b. Post-MI<br>c. 59<br>d. NR	a. CAP-CR platform used a smartphone application and step-counter for health and exercise monitoring, and delivery of motivational and educational materials to participants via text messages and preinstalled audio and video files. Mentors provided feedback on progress of goals set via weekly telephone consultations.<br>b. 6 weeks.	Quantitative (survey questionnaire)	Intra-trial
Varnfield et al. (2014)/ Australia	a. RCT<br>b. Investigate if CAP-CR is effective in improving CR use in post-MI patients	a. *N* = 53<br>b. Post-MI<br>c. 54.9 ± 9.6<br>d. 9.4%		Quantitative (captured usage data)	Intra-trial
Wang et al. (2020)/ China	a. RCT<br>b. Determine whether a WeChat-based intervention could be an effective way to improve secondary prevention adherence after CABG	a. N = 81<br>b. CABG<br>c. 64 ± 8.7<br>d. 21%	a. Participants accessed weekly education articles and were encouraged to upload BP and blood tests data onto the WeChat platform. Two cardiologists and a trained nurse reviewed participants' data and enquiries, and provided feedback as required. A cardiologist conducted online medication reviews every 4weeks.<br>b. 6months	Quantitative (captured usage data)	Intra-trial
Worringham et al. (2011)/ Australia	a. Feasibility study<br>b. Evaluate the feasibility of a remotely-monitored exercise-based CR in cardiac patients	a. *N* = 6<br>b. CHD or CRV<br>c. 53.6 (42 – 67)<br>d. 20%	a. The smartphone captured data from a single lead ECG trace, HR activity monitor, & walking speed, elapsed distance, and patient location via GPS receiver to be viewed on a secure server in real-time. Voice only mobile phone provided for pre- and post-session or routine/emergency contact during an exercise session if needed.<br>b. 6 weeks	Quantitative (captured usage data, questionnaire & semiqualitative survey)	Pre-trial
Yu et al. (2020)/ China	a. RCT<br>b. Evaluate the effectiveness and feasibility of using a smartphone-based application to improve medication adherence in patients after CABG	a. N = 501<br>b. CABG<br>c. 57.41 ± 8.99<br>d. 13.6%	a. Heart Health smartphone-based application automatically reminded the participants when it was time to take each medication, and participants could confirm that the medicine had been taken via the app. Educational readings on secondary preventive cardiac care were provided.<br>b. 6 months	Quantitative (captured usage data & survey questionnaire)	Combination of intra- & post-trial
Yudi et al. (2020)/China	a. RCT<br>b. Evaluate the efficacy of smartphone-based CR program on exercise	a. N = 501<br>b. CABG<br>c. 57.41 ± 8.99<br>d. 13.6%	a. Exercise prescription and real-time feedback by the smartphone's accelerometer feature, dynamic tracking of cardiovascular RF, assessment of dietary habits, heart health and secondary prevention pharmacotherapy, as well as interactive and personalized feedback (5x/week) and support (as required).	Quantitative (captured usage data)	Intra-trial

This is a Multimedia Appendix to a full manuscript published in the J Med Internet Res. For full copyright and citation information see https://www.jmir.org/2022/1/e34657

	capacity and cardiac RF modification.		b. 8 weeks		
Zutz et al. (2007)/ Canada	a. Pilot RCT<br>b. Assess the feasibility and safety of using the Internet as a medium for delivery of an interactive vCRP	a. N = 8<br>b. MI, PCI, CABG<br>c. 58 ± 4<br>d. 12.5%	a. vCRP (password-protected) included weekly education, one-on-one chat sessions with the program nurse case manager, exercise specialist and dietitian, monthly ask-an expert group chat session. Exercise data from HR monitors were uploaded on to the vCRP. Participants also entered their weight, BP, and BG (if diabetic) for review.<br>b. 12 weeks	Quantitative (captured usage data) & Qualitative (semi-structured interviews)	Pre-trial
Lear et al. (2014)/ Canada	a. RCT<br>b. Test the clinical effectiveness of vCRP.	a. *N* = 78<br>b. ACS or CRV<br>c. 61.7 ± 10.4<br>d. 15.4%	a. The vCRP included on-line intake forms (medical, risk factor and lifestyle), scheduled one-on-one chat sessions with nurse, exercise specialist and dietician, weekly education sessions, and data capture for exercise and blood test results.<br>b. 16 weeks	Quantitative (captured usage data)	Intra-trial
Banner et al. (2015)/ Canada	a. Descriptive qualitative study<br>b. Explore the acceptability and uptake of the vCRP program.	a. *N* = 22<br>b. ACS or CRV<br>c. NR<br>d. NR		Qualitative (semi-structured interviews)	Post-trial

RCT, Randomized controlled trial; CR, Cardiac rehabilitation; CHD, Coronary heart disease; RF, Risk factor; PA, Physical activity; PCI, Percutaneous coronary intervention; CABG, Coronary artery bypass graft; AMI, Acute myocardial infarction; SUS, System Usability Scale; SMART-CR/SP, Smartphone-based-Cardiac Rehabilitation/Secondary Prevention; BP, Blood pressure; HR, Heart rate; HBCTR, Home-based cardiac telerehabilitation; SMS, Short message service; ACS, Acute coronary syndrome; REMOTE-CR, Remotely monitored exercise-based cardiac rehabilitation; CRV, Coronary revascularization; CAP-CR, care assessment platform-cardiac rehabilitation; ECG. Electrocardiogram; GPS, Global positioning system; vCRP, Virtual Cardiac Rehabilitation Program; BG, Blood glucose

This is a Multimedia Appendix to a full manuscript published in the J Med Internet Res. For full copyright and citation information see https://www.jmir.org/2022/1/e34657

Table S4. Characteristics of Included HBCTR Programmes

	Modes of delivery							HBCTR Features					Core Components of HBCTR					
	Website	Mobile application	Text message	Phone call	Email	Tele-monitoring	Video calls	Design	Testing	Training	Technology support	Data privacy	Patient assessment	Exercise training	Dietary management	Risk factor management	Medication adherence	Psychosocial support
Banner et al. 2015	Present				Present	Present		Present	Present	Present		Present	Present	Present	Present	Present		
Devi et al. 2014	Present				Present	Present		Present		Present		Present	Present	Present	Present	Present		Present
Ding et al. 2021		Present		Present		Present			Present	Present	Present	Present	Present	Present	Present	Present		
Dorje et al. 2019		Present	Present			Present			Present	Present		Present	Present	Present	Present	Present	Present	Present
Fang et al. 2019		Present		Present		Present			Present	Present			Present	Present	Present	Present	Present	Present
Harzand et al. 2018		Present	Present	Present					Present				Present	Present	Present	Present	Present	
Higgins et al. 2017	Present								Present				Present	Present	Present	Present	Present	Present
Rawstron et al. 2018		Present	Present			Present				Present	Present	Present	Present	Present				
Song et al. 2020		Present	Present	Present		Present				Present	Present		Present	Present				
Varnfield et al. 2014		Present	Present			Present	Present			Present	Present		Present	Present	Present	Present	Present	Present
Wang et al. 2020		Present						Present					Present	Present	Present	Present	Present	Present
Worringham et al. 2011		Present		Present		Present			Present			Present	Present	Present				
Yu et al. 2020		Present							Present	Present		Present	Present			Present	Present	
Yudi et al. 2020		Present	Present			Present				Present			Present	Present	Present	Present	Present	

■ Present □ Not Present

This is a Multimedia Appendix to a full manuscript published in the J Med Internet Res. For full copyright and citation information see https://www.jmir.org/2022/1/e34657

Table S5. Technology Acceptance Outcomes in Included Studies

Author (Year)<br>Technology Acceptance Construct	Technology Acceptance Outcomes
**Banner et al. (2015)**	
External Variables	Participants were satisfied with the virtual format as it was accessible, convenient negated the need for travel and provided easy access to key healthcare professionals. Lack of time, infrequent access to the Internet or computer, lack of motivation, and poor computer literacy influenced levels of engagement. Challenges related to the use of the computer were largely isolated to the initial set up and were resolved with greater familiarity or direct support. Ongoing surveillance from healthcare providers, as well as support for self-management activities, helped participants to adhere to their recommended program.
Usability	Feedback from interviews reported that the uploading of HR data onto the VCRP website was easy to perform
Utility	Participants reported greater awareness and motivation to manage their health condition and maintain a healthy diet, undertake regular exercise with suitable intensity, and to monitor their health condition appropriately. Participants reported that they felt more accountable for their progress and confident in their recovery.
Acceptability	NR
**Devi et al. (2015)**	
External Variable	Participants valued convenience of the programme as there was no time or location restrictions; tailored information; communication with a healthcare professional during the program; and goal setting with an online exercise diary increased motivation. Participants reported barriers were perceived lack of time due their family responsibilities and employment; timing of programme introduction too late after diagnosis and in the winter made it difficult to exercise outdoors; preconceptions about the programme being designed and more suitable for a younger age group. Participants reported that self-motivation, seriousness and honesty were required as the programme was carried out independently
Usability	NR
Utility	Participants reported feeling more confident and encouraged to try different ways to engage in physical activity and reported decreased anxiety and improved psychological well-being. Participants felt empowered and more in control and better able to manage their stress and symptoms.
Acceptability	NR
**Ding et al. (2021)**	
External Variables	NR

This is a Multimedia Appendix to a full manuscript published in the J Med Internet Res. For full copyright and citation information see https://www.jmir.org/2022/1/e34657

Usability	System Usability Scale (SUS) median score was 82.5 (IQR 65.0, 90.0); 82% of participants agreed that the system was easy to use and that people can learn to use quickly, 71% agreed that the functions were well integrated; 71% disagreed that the system use required technical support and 81% disagreed that a lot of learning was required prior to use; 13% found the system unnecessarily complexed.
Utility	82% of participants (n = 14) reported that the system motivated them to be physically active and helped them to achieve physical activity recommendations; 35% (n = 6) agreed that they walked and exercised more than they previously had
Acceptability	NR
**Dorje et al. (2019)**	
External Variables	NR
Usability	NR
Utility	Participants who agreed/strongly agreed that: SMART-CR/SP was helpful to me 100% (n = 125); Eat more healthily after receiving SMART-CR/SP 98% (n = 122); Increased physical activity frequency and intensity 96% (n = 100); Increased medication adherence 98% (n = 123); Reduced outpatient clinic visits 78% (n = 98)
Acceptability	NR
**Fang et al. (2016)**	
External Variables	Participant reports for accepting HBCTR: Make life safer and independent 28.3% (n = 49); Being able to self-monitor physical conditions daily 25.4% (n = 44); Automatic emergency alerts 23.1% (n = 40); Having regular professional rehabilitation 16.8% (n = 29); Assurance to family members 6.4% (n = 11)<br>Participant reports for rejecting HBCTR: Cumbersome operation 34.3% (n = 37); Unnecessary cardiac rehabilitation procedure 19.4% (n = 21); Unreliable technology 16.7% (n = 18); Inaccurate monitoring information 13.0% (n = 14); Needing specialized coaching 9.3% (n = 10); Concerns for safety 4.6% (n = 5); Breach of privacy 2.8% (n = 3)
Usability	NR
Utility	NR
Acceptability	59.3% (n = 89) reported willingness to participate in HBCTR
**Harzand et al. (2018)**	
External Variables	NR
Usability	Participants reported that the platform was "not hard to use" and "at times fun"

This is a Multimedia Appendix to a full manuscript published in the J Med Internet Res. For full copyright and citation information see https://www.jmir.org/2022/1/e34657

Utility	Program helped participants to set goals and they reported that it was “nice to have reminders” on the platform
Acceptability	NR
**Higgins et al. (2017)**	
External Variables	Qualitative data indicated that patients appreciated the variety inherent in the range of modules, although there were concerns regarding the feasibility of completing all the modules and wanted the program earlier in their recovery before they had returned to usual activities and work. Participants wanted the program to be more interactive in terms of both tailoring entry into the relevant modules according to the user profile to avoid missing them and also creating opportunities for users to interact with each other. 24% reported that the program was too simplistic, and would be more appropriate for patients with lower health literacy.<br>Greater attention to strengths and meaning was also suggested to help align behavior change goals with life goals. One shortcoming of the program was in the neglect of the area of ‘‘death anxiety.’’
Usability	85.7% of participants agreed that the system was easy to navigate, 76.2% of participants liked the layout of the system and 90.5% disagreed that the system was too complicated; Qualitative data from interviews reported that participants found the system was well set out, easy to understand and follow, information presented was not overpowering, and the graphics were good
Utility	76% of participants agreed that the system gave them strategies to help change their lifestyle; 62% agreed that the program helped them make lifestyle changes and increased their confidence to cope with health problems; 48% agreed that the program helped them to get more motivated; 48% reported that the program gave them strategies to help manage their emotions. Qualitative data revealed that the program was seen as something which would help us long term, appeared to reinforce and support behavior change and that the normalization of emotional experience was an important aspect of the program for participants.
Acceptability	NR
**Rawstorn et al. (2018)**	
External Variables	97% (n = 65) participants felt well supported during the program; 32.8% (n = 22) would have valued additional intra-program support (eg, telephone follow-up in addition to real-time monitoring, more intensive coaching); 35.8% (n = 24) would have valued app functionality that facilitated social interaction with telerehabilitation peers; 98.5% (n = 66) valued universal accessibility of the program. 89.5% (n = 60) were satisfied with the individualized exercise prescription and 94% (n = 63) liked the real-time monitoring/ coaching from exCR specialists as they facilitated confidence, motivation to adhere to prescribed exercise intensity levels, and provided reassurance for participants initiating exercise during post-acute recovery. Real-time self-monitoring 95.5% (n = 64), post-exercise performance review 89.6% (n = 60), post-exercise message review 83.6% (n = 56), goal setting 68.7% (n = 46), and goal achievement feedback 68.7% (n = 46) were liked features as they improved self-awareness and facilitated progress evaluation. Participant-reported opportunities to improve REMOTE-CR included real-time participant-to-exCR specialist communication and music/radio integration, extending real-time monitoring operating hours, and optimizing the app user interface for legibility during exercise. Factors that commonly affected engagement with REMOTE-CR included work/family commitments and motivation. Only 1 participant reported age-related usability barriers.

This is a Multimedia Appendix to a full manuscript published in the J Med Internet Res. For full copyright and citation information see https://www.jmir.org/2022/1/e34657

Usability	Wearable sensor: 98.5% (n = 66) agreed it was easy to use and 97% (n = 65) agreed it was comfortable to wear<br>Smartphone application: 79.1% (n = 53) agreed it was easy to use and 86.6% (n = 58) agreed it was easy to understand and reliable (n = 44; 65.7%)
Utility	REMOTE-CR facilitated confidence and motivation to adhere to prescribed exercise intensity levels, and provided reassurance for participants initiating exercise during post-acute recovery. Satisfaction with offline self-monitoring, goal setting, and goal achievement feedback were also high as they improved self-awareness and facilitated progress evaluation.
Acceptability	86.6% (n = 58) of participants indicated they would choose REMOTE-CR if it was available via usual cared predominantly due to enhanced accessibility, flexibility, and convenience
**Song et al. (2020)**	
External Variables	91.7% (n = 44) of the participants were satisfied with the content and frequency of feedback; others were not completely satisfied with the feedback because they were not proficient in uploading information even with guidance of researchers.
Usability	NR
Utility	NR
Acceptability	NR
**Varnfield et al. (2014)**	
External Variables	Reasons for not using the Wellness Diary Connected were mainly not having a computer or internet access. 91% of the participants reported that phone consultations with mentors motivated them to meet their goals.
Usability	Participants reported that the modalities of the smartphone app were easy to use.
Utility	Only two participants indicated that the application was not useful for self-management.
Acceptability	NR
**Worringham et al. (2011)**	
External Variables	Over 80% of completed sessions had no technical problems. Of the remaining sessions, intermittent signal interruption attributable to poor mobile phone coverage was the most common problem, followed by loss of battery power (Table 1).
Usability	Ease of use rating: 4.8 (95% CI, 4.6–5.0), where 4= quite easy and 5 =very easy; Frequency of technical problems rating: 4 (95% CI, 4.1–4.9), where 4 =less than once a week and 5= never; Equipment took an average of 3.9 minutes to put on and 3.0 minute to remove

This is a Multimedia Appendix to a full manuscript published in the J Med Internet Res. For full copyright and citation information see https://www.jmir.org/2022/1/e34657

Utility	NR
Acceptability	NR
**Yu et al. (2020)**	
External Variables	NR
Usability	NR
Utility	15.0% of participants thought the application was very useful, and more than half of the participants thought the application was of little use or useless.
Acceptability	Approximately 81.3% of participants said they might continue to use the application
**Zutz et al. (2007)**	
External Variables	Participants found the interactive components (62.5%), ability to view personal records (53%), and the scheduled chat sessions (50%) to be most effective and technology-based aspects, such as the server connection (75%), required the most improvement. in
Usability	The uploading of the "heart rate" was pretty easy
Utility	Participants reported that nutrition portion was extremely valuable and a constant reminder of in decision-making; all physiotherapy sessions were extremely useful as well; chat sessions helped answer questions; the BP and exercise charts helped to visualize progress and set goals.
Acceptability	NR

This is a Multimedia Appendix to a full manuscript published in the J Med Internet Res. For full copyright and citation information see https://www.jmir.org/2022/1/e34657
